# Regulatory Systems for Prevention and Control of Rabies, Japan

**DOI:** 10.3201/eid1409.070845

**Published:** 2008-09

**Authors:** Hiromi Takahashi-Omoe, Katsuhiko Omoe, Nobuhiko Okabe

**Affiliations:** National Institute of Science and Technology Policy, Tokyo, Japan (H. Takahashi-Omoe); Iwate University, Iwate, Japan (K. Omoe); National Institute of Infectious Diseases, Tokyo, Japan (N. Okabe)

**Keywords:** rabies, laws, government regulations, synopsis

## Abstract

Japan is one of the few rabies-free countries. Although 3 imported cases of human rabies were seen in 1970 and 2006, no other cases have been reported for ≈50 years. The elimination of rabies in Japan is attributed to not only its geographic isolation but also to effective prevention and control measures, such as registration and vaccination of domestic dogs, required quarantine of susceptible imported animals, and national plans of action based on scientific research. Countermeasures against rabies have been upgraded; an improved management system for domestic dogs under the amended Enforcement Regulations of the Rabies Prevention Law has been in effect since April 2007. The latest regulatory systems for preventing and controlling rabies provide an effective model for elimination of the disease worldwide.

## Abstract 2

Japan’s systems provide an effective model for elimination of rabies worldwide.

Rabies is a severe zoonotic viral disease that kills ≈55,000 persons annually in many countries of Africa and Asia (*1*). Because of the lack of specific and effective medical care for persons with clinical rabies (*2*–*4*), many countries have taken various measures to prevent and control rabies in animals. Rabies-free countries and territories are limited to islands such as Japan and New Zealand and to parts of northern continental Europe (*5*). Japan has been free of rabies for ≈50 years; the last cases of human and animal rabies were reported in 1954 and 1957, except for 3 imported cases of human rabies in 1970 and 2006 (Table 1) (*6*–*9*).

**Table 1:** Annual transition of rabies outbreaks in Japan*

Year	No. cases in dogs (cats)	No. cases in humans	No. cases in livestock	Remarks
1945	94 (2)	1	19	
1946	24 (1)	1	5	
1947	37	17	1	
1948	141 (1)	45	2	
1949	614 (10)	76	2	
1950	867 (29)	54	12	Enforcement of Rabies Prevention Law
1951	319 (3)	12	18	Enforcement of Domestic Animal Infectious Diseases Control Law
1952	232	4	1	
1953	176	3	4	
1954	98	1	No data	
1955	23	0	No data	
1956	6	0	No data	
1957	0 (1)	0	No data	
1970	0	1	0	Imported case (returning traveler from Nepal)
2006	0	2	0	Imported cases (returning travelers from the Philippines)

*Data from the aggregate calculation by the Ministry of Health, Labour and Welfare (*6*).

In Africa and Asia, human rabies is contracted primarily from rabid dogs. However, several wild animal species, including bats and foxes, are carriers and vectors for rabies and related viruses in the genus *Lyssavirus* (*10**,**11*). Although lyssaviruses have been isolated from wild animals in many countries, in Japan such viruses have not been reported in any animals during the past decade (*12*).

Japan has long been free of rabies because it is separated by water from countries in which the disease is endemic and because it has successfully managed rabies prevention and control. Management techniques include registration and vaccination of domestic dogs, legal regulations to quarantine susceptible imported animals, and national plans of action based on scientific research. Nevertheless, outbreaks of animal or human rabies, such as the cases in 2006, and recent increases in the international movement of people and animals have raised concerns.

A further cause for concern is the decreasing percentage of vaccinated domestic dogs among all registered dogs in Japan. According to data reported in 2006 (*13*), 4,910,047 (74%) of 6,635,807 registered domestic dogs were vaccinated. However, because the percentage of registered dogs is assumed to be ≈50% of the total number of dogs in Japan, immunization coverage may actually be <40% (*14*).

Because of the increasing risk for domestic and international rabies outbreaks, Japanese central and local governments, in conjunction with coalitions of public health specialists such as veterinarians, physicians, and researchers, have developed several preventive measures. We present the country-level management systems in Japan, focusing on the latest legal regulations and plans of action. We believe that Japan’s approach to preventing and controlling rabies is an effective model for the elimination of rabies throughout the world.

## Legal Framework

**Figure 1:**
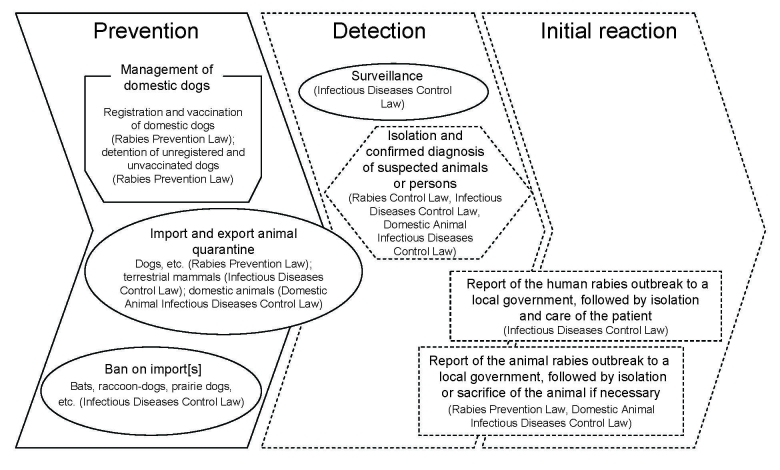
Regulatory framework for preventing and controlling rabies in Japan. Under 3 laws, countermeasures against rabies are divided into prevention, detection, and initial reaction. Infectious Diseases Control Law means Law Concerning the Prevention of Infectious Diseases and Medical Care for Patients with Infectious Disease. Solid and dashed lines show ordinary and emergency countermeasures, respectively.

Preventive measures against human and animal rabies in Japan are stipulated under 3 laws: the Rabies Prevention Law (no. 247, August 1950, and amended law no. 160, December 1999); the Domestic Animal Infectious Diseases Control Law (no. 166, May 1951, and amended law no.102, October 2005); and the Law Concerning the Prevention of Infectious Diseases and Medical Care for Patients with Infectious Diseases (Infectious Diseases Control Law; no. 114, October 1998, and amended law no. 30, May 2008) (Figure 1) (*15*–*18*). Under these laws, substantive efforts to prevent and control rabies have been adopted by central and local governments, relevant ministries, various concerned bodies, veterinarians, physicians, and researchers.

The measures enforced under these laws are divided into 2 categories: 1) day-to-day measures, such as the registration and vaccination of domestic dogs, and 2) export and import quarantine of animals that are susceptible to rabies. The former is managed primarily by the Ministry of Health, Labour and Welfare of Japan (MHLW) and the public health departments of local governments, and the latter is managed by the Ministry of Agriculture, Forestry and Fisheries of Japan (MAFF), and quarantine stations. The usual preventive measures, emergency measures in case of an outbreak of human and/or animal rabies and related plans of actions, are promulgated in these laws. The essential features of these regulatory systems are described below.

### Animal Rabies Control under the Rabies Prevention Law

The regulatory system to control rabies in pets and wild animals is based on the Rabies Prevention Law (*15*). The objectives of the law are to improve public health and contribute to public welfare by preventing outbreaks of rabies, controlling its spread in the event of an outbreak, and therefore eliminating the disease. The animals targeted under this law are dogs, cats, and other animals (e.g., raccoons, foxes, skunks) that have a high potential to infect humans.

This law focuses particularly on the development of daily administrative systems for domestic dogs (*19*). Under these systems, all dog owners are required to register their dogs and have them vaccinated against rabies. Owners must register their dog with the head of the nearest local government once during the animal’s lifetime; after registration, the dog must wear a license tag. Regarding vaccination, dog owners must have their dog vaccinated against rabies once a year. After vaccination, the owner must take the vaccination certificate from the veterinarian who administered the vaccine to the head of the nearest local government, where they will receive a certification tag that the dog must wear. Local governments are responsible for managing registration and vaccination of dogs and for assigning veterinarians who capture and detain unregistered or unvaccinated dogs.

To strengthen this management structure, 2 measures have been taken. One is the amended Enforcement Regulations of the Rabies Prevention Law, enacted in April 2007 (MHLW ordinance no. 52, September 1950, and amended ordinance no. 17, March 2007), which provides improved standards for licensing and certification of vaccinated dogs. The amendment offers 2 improvements: 1) the miniaturization of the license and vaccination certification tags so that they can be attached to smaller dogs and 2) the ability of local governments to choose the shape of the license and certification tags (*20*). The other measure is the approach by the Japan Veterinary Medical Association (JVMA) to strengthen rabies control (*14*). JVMA encourages dog owners to keep their animals vaccinated against rabies because actual immunization coverage is assumed to be <40% in Japan (*14*); nevertheless, the World Health Organization (WHO) recommends immunization coverage of at least 70% to control canine rabies in areas where the disease is endemic (*21*). Additionally, JVMA asks for public understanding and cooperation regarding rabies vaccination.

In addition to the management of domestic dogs described above, the law stipulates import and export quarantine for animals that are susceptible to rabies. The quarantine system, which is based on the latest scientific diagnostic knowledge and which makes use of examples from the UK quarantine system entitled the Pet Travel Scheme (*22*), has been enforced since November 2004 under the Regulations for Import and Export Quarantine of Dogs and Other Designated Animals (MAFF ordinance no. 68, November 1999, and amended ordinance no.75, November 2004) (Quarantine Regulation by MAFF). Under this system, dogs, cats, raccoons, foxes, and skunks are identified as animals subject to quarantine in a MAFF Animal Quarantine Service facility. The quarantine detention period is from 12 hours to 180 days, depending on the status of rabies outbreaks in the animal’s region of origin and preparation of the required certification (Table 2). Detention for 12 hours is applicable for dogs, cats, raccoons, foxes, and skunks imported directly from rabies-free regions (designated regions) and dogs and cats vaccinated and inspected in regions other than designated regions. Detention for 180 days is required for all raccoons, foxes, and skunks imported from regions other than designated regions. Further details concerning the quarantine system, such as forms for notification and certification, can be found in the practical guide by MAFF (*23*,*24*).

**Table 2:** Detention period for quarantining imported animals under the Rabies Prevention Law

Animals	Imported from designated regions (rabies-free regions)*			Imported from other regions	
	Detention within 12 h	Detention for >12 h		Detention within 12 h	Detention for >12 h
Dogs and cats	Necessary procedures before import: prior notification concerning import† attached by a health certificate type A. Contents of type A certificate: 1. Individual identification by microchip‡; 2. residency in the exporting country for at least 180 d immediately before shipment to Japan, or since birth, or continuous residency in the exporting country since being directly imported to Japan; 3. no case of rabies in the exporting country for at least 2 y before exporting the animal; 4. clinical examination showing rabies-free (dog and cat) and leptospirosis-free (dog) proof	Extended quarantine period up to 180 d in the case of omissions in prior notification† attached by a certificate type A		Necessary procedures: prior notification concerning the import† attached by a health certificate type B. Contents of type B certificate: individual identification by microchip,‡ rabies vaccination using inactivated vaccines at least twice, rabies serologic test,§ a wait of at least 180 d between the date of blood sampling (day 0) and the date of arrival of an animal in Japan	Extended quarantine period up to 180 d in the case of omissions in prior notification† attached by a certificate type B
Raccoons, foxes, skunks	Necessary procedures before import: prior notification concerning the import† attached by a health certificate type C. Contents of type C certificate: individual identification by microchip,‡ rabies vaccination using inactivated vaccines at least twice, rabies serologic test,§ clinical examination showing rabies-free proof	Extended quarantine period up to 180 days in the case of omissions in prior notification† attached by a certificate type C			Necessary procedures: prior notification†, individual identification by microchip‡, clinical examination. Fixed quarantine period (180 d)

*Designated regions are Taiwan, Iceland, Sweden, Norway, United Kingdom (only Great Britain and Northern Ireland), Australia, New Zealand, Fiji Islands, Hawaii, and Guam. All regions were designated on June 7, 2005. †When trying to import dogs or cats, the person must submit the advance notification described below to the Animal Quarantine Service, which has jurisdiction over the person’s intended port of arrival, at least 40 d before arrival in Japan. Notification items include name, address, and contact number of the person submitting the notification; breed of dog/cat; number of animals; intended use; country of export; date and place of import; name and address of consignee/consigner; export location/destination; and individual identification data. ‡An International Organization for Standardization–compliant microchip (ISO11784 and ISO11785) should be used. If another type of microchip is used, a special reader for the microchip is needed. §For the rabies serologic test, the neutralizing antibody titration test against rabies is necessary after the second vaccination. The test must be carried out by a laboratory designated by the Minister of Agriculture, Forestry and Fisheries of Japan. Test results must be >0.5 IU/mL.

### Animal Rabies Control under the Domestic Animal Infectious Diseases Control Law

**Figure 2:**
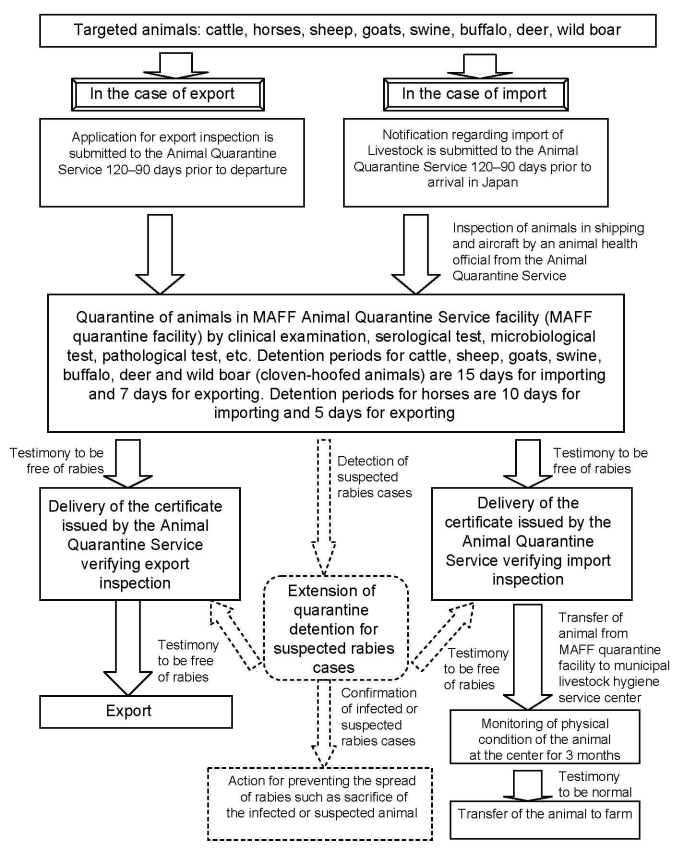
Flowchart of the inspection for rabies infection for importing and exporting animals under the Domestic Animal Infectious Diseases Control Law. The figure is based on our interpretation of data from reference (*25*). Dashed lines show emergency countermeasures. MAFF, Ministry of Agriculture, Forestry and Fisheries.

The regulatory system to control rabies in livestock is based on the Domestic Animal Infectious Diseases Control Law (*18*). The law has been implemented to domestically and internationally promote the livestock industry by preventing the outbreak and spread of infectious diseases in domestic animals. Under this law, rabies in cattle, horses, sheep, goats, swine, buffalo, deer, and wild boars is designated as a “domestic animal infectious disease (infectious disease obligated to report).” Livestock intended for import and export are quarantined to prevent outbreaks and spread of rabies not only in Japan but also in other countries. As shown in Figure 2 (*25*), the quarantine detention period for the above animals differs according to species (cloven-hoofed animals or horses) and whether the animals are being imported or exported. For imported animals, double inspections have been implemented to detect 100% of infected animals before they are transferred to a farm. One inspection is a microbiologic test conducted at the MAFF Animal Quarantine Service facility; the other is the monitoring of physical condition of animals at the municipal livestock hygiene service center. Details on the quarantine system can also be found in the information manual of the animal quarantine system by the Japan External Trade Organization (*26*).

When rabies is suspected or confirmed in livestock, the diagnosing veterinarian or animal owner is required to report the case immediately to a prefectural or city governor through the director of the nearest animal public health center. It is also a legal requirement that the animal be isolated, and euthanized if necessary.

### Animal Rabies Control under the Notification System for the Importation of Animals

To prevent the invasion of infectious diseases, including rabies, through animals imported into Japan, the Notification System for the Importation of Animals, authorized by the Infectious Diseases Control Law, has been in force since September 2005 (*27*). With respect to the use of quarantine to control rabies, the system requires terrestrial mammals being exported to be accompanied by health certificates declaring the animals to be free of the disease; the certificates are issued by government authorities of the exporting country. Target mammals include not only animals for distribution and exhibition in Japan but also animals for personal possession as pets; they do not include animals that have already been quarantined under the Rabies Prevention Law or the Domestic Animal Infectious Diseases Control Law or animals whose importation is banned by the Infectious Diseases Control Law (Chinese ferret badgers; bats; raccoon dogs; masked palm civets; prairie dogs; *Mastomys natalensis;* and all monkeys except those used for experimentation, research, and exhibition in Japan). Therefore, the notification system plays a complementary role in the quarantine specified under the Rabies Prevention Law and the Domestic Animal Infectious Diseases Control Law. Animal species that are quarantined under the above 3 laws are shown in Table 3.

**Table 3:** Animals subject to quarantine and/or examination for rabies before importation into Japan

Law or regulation	Animals subject to quarantine	Animals requiring a health certificate	Animals banned from importation
Rabies Prevention Law (Regulations for Import and Export Quarantine of Dogs and Other Designated Animals*)	Dogs, cats, raccoons, foxes, skunks		
Domestic Animal Infectious Diseases Control Law	Cattle, horses, sheep, goats, swine		
Law concerning the Prevention of Infectious Diseases and Medical Care for Patients with Infectious Diseases	Monkeys used for research and exhibition, under specified conditions only		Chinese ferret badgers, bats, raccoon dogs, masked palm civets, prairie dogs, *Mastomys natalensis* rats, monkeys except those to be used for research or exhibition
Notification System for the Importation of Animals†		Terrestrial mammals except for Artiodactyla (e.g., cattle, sheep, goats); Perissodactyla (e.g., horses); Lagomorpha (e.g., rabbits); dogs, cats, raccoons, foxes, skunks, monkeys	

*This regulation is authorized by the Rabies Prevention Law. †This regulation is authorized by the Law Concerning the Prevention of Infectious Diseases and Medical Care for Patients with Infectious Diseases.

### Human Rabies Control under the Infectious Diseases Control Law

Japan’s regulatory system for human rabies control is based on the Infectious Diseases Control Law (*16*,*17*). The objective of the law is to control outbreaks of infectious diseases, including zoonoses, and to prevent the spread of these diseases in humans. The law targets ≈100 kinds of infectious diseases (*28*) and stipulates the medical care for patients affected by the diseases to promote, improve, and upgrade public health in Japan. Regarding human rabies, the law requires reporting of disease cases promptly after diagnosis. In the instance of well-defined or suspected human rabies, the diagnosing physician must report the case immediately to the director of the nearest public health center, who will then forward the report to the local government.

## National Standards for Rabies Control

The 2001 Guidelines on Rabies Countermeasures (MHLW Notification, November 2001, and supplement, January 2003) have been put into practice as the standard for preventing and controlling rabies according to the above laws (*29**–**31*). The guidelines are described in a comprehensive handbook for addressing an outbreak or suspected outbreak of rabies in Japan; they establish measures to guide government, medical, and other related institutions in taking suitable initial actions. These measures are based on a number of documents: Laboratory Techniques in Rabies, published by WHO (*32*); Laboratory Methods for Detecting Rabies, by the US Centers for Disease Control and Prevention (*33*); Rabies Contingency Plan in Hawaii (*34*); and Memorandum of Rabies, Prevention and Control, by the UK Department of Health (*35*). The latest guidelines include a supplement concerning the response to the increasing risk for rabies infection through rabid animals and the status of rabies outbreaks in the world.

The 2001 Guidelines on Rabies Countermeasures base specific countermeasures against suspected cases of animal and human rabies on the location of cases. These countermeasures are divided into 7 patterns to facilitate a quick response, depending on the situation (*29*–*31*). Each pattern involves role sharing between the Japanese central and local governments; networking among affected organizations such as veterinary hospitals, animal control facilities, and medical institutions; measures for dealing with people and animals that might come into contact with rabid animals; and specific examination procedures.

The 2 cases of human rabies in 2006 (*7*–*9*) were stringently controlled according to the 2001 Guidelines on Rabies Countermeasures, in terms of the initial response to a rabies outbreak and medical practice; the patients, however, died of the disease. It was possible to make a rapid, definitive diagnosis by detecting the rabies virus gene on days 2–3 (first case) (*7*,*8*) and on day 2 (second case) (*8*,*9*). For the first case, the health professional who treated the patients in the hospital worked smoothly with local governments, the National Institute of Infectious Diseases, and MHLW to enable urgent health advice to be given quickly to the related organizations such as quarantine stations and local governments on day 4. Concerning the second case, effective countermeasures published in an overseas case report and manual were also applied. The patient was isolated strictly, following the recommendations of the Centers for Disease Control and Prevention manual (*36*); in addition to isolation, the patient received the same medical care as that given to a patient who had survived (*2*,*3*).

## Conclusion

Japan has successfully eliminated rabies because of its geographic isolation and because of the systematic management of susceptible animals and humans under the relevant laws and regulations. These effective preventive measures enforced under the regulatory systems serve as a model for elimination of the disease worldwide.

As a remaining task for controlling rabies in Japan, internal and international rabies surveillance should be maintained or increased in the years ahead. Previous reports suggest that no rabies or other lyssaviruses have been detected in animals during the past decade in Japan (Table 1) (*6*,*12*); however, surveillance of domestic and wild animals that are possible hosts for infection in Japan should be followed up continuously because of the <40% immunization coverage of dogs (*14*).

In addition to domestic countermeasures against rabies, border control measures to eliminate possible importation of animal or human rabies cases should be strengthened. Regardless of quarantine system, which theoretically makes it possible to eliminate the entry into Japan of an animal infected with rabies or other lyssavirus, the risk for rabies in Japan is believed to be rising (*14*). This belief is because the international movement of people and animals is increasing and the illegal importation of rabid animals remains a possibility, as does the immigration of people who are unaware that they have been infected with rabies or other lyssaviruses. To eliminate these possibilities, it is necessary to control such animals thoroughly by stringent import quarantine and to highlight the risk for rabies infection to Japanese nationals, who tend to consider the disease to have been eradicated in Japan and therefore may be less vigilant than necessary.

Moreover, surveillance of rabies and lyssavirus infections in wild animals is needed for further rabies control internationally because several wild animal species are recognized as wildlife carriers of rabies and lyssaviruses worldwide. In recent years, our understanding of the epidemiology of rabies and lyssaviruses has changed substantially as a result of improved molecular approaches to virus variant identification and improved epidemiologic analysis techniques for rabies and lyssavirus infections. However, epidemiologic data from Asian countries have not been sufficiently collected and analyzed (*37*). Japan must survey the distribution of rabies and lyssavirus infections in nearby Asian countries from the standpoint of international cooperation in terms of control of rabies and improvement of the import quarantine system. Thus, Japan needs to promote surveillance of rabies and lyssavirus infections internationally, focusing on not only dogs but also other animals, especially wild animals. As a surveillance attempt, scientists in Japan and other Asian countries have epidemiologically and phylogenetically examined domestic and wild animals living in Asian countries (Appendix) (*38*,*39*) and discussed a measure for developing a new type of rabies vaccine based on the surveillance data (*39*). Because more surveillance and analysis data regarding rabies and lyssaviruses diseases in Asian countries will be published, a network responsible for amassing and systematizing the data provided by scientists should be established by a coalition of not only scientists but also of governments and healthcare professionals, such as veterinarians and physicians, in Asian countries. Creating a new network for the control of rabies and lyssavirus diseases is timely, is of global interest, and represents a further contribution to the successful elimination of the diseases around the world.

## Supplementary Material

Appendix Regulatory Systems for Prevention and Control of Rabies, Japan
