# Supplementary material for: Regulatory Systems for Prevention and Control of Rabies, Japan
Source: Emerg Infect Dis. 2008 Sep;14(9):1368–74. doi: 10.3201/eid1409.070845 (PMC2603086; doi:10.3201/eid1409.070845)
Supplement: Appendix — Regulatory Systems for Prevention and Control of Rabies, Japan [file 07-0845_app-s1.pdf]

Publisher: CDC; Journal: Emerging Infectious Diseases
Article Type: Synopsis; Volume: 14; Issue: 9; Year: 2008; Article ID: 07-0845
DOI: 10.3201/eid1409.070845

# Appendix:
# Regulatory Systems for Prevention and Control of Rabies, Japan

## The Study on International Surveillance of Rabies and Lyssavirus Infections Caused by Wild Animals (Tentative English Ttranslation)

This study (*1*) is in progress under the Grants for Health Science from the Ministry of Health, Labour and Welfare of Japan (MHLW). The research fund is 10,000,000 yen, and the study duration is 3 years (FY 2006–2008). Abstract of the research is currently available on the Internet (release date April 20, 2007).

## Purpose of the Study

Japan has been free of rabies, but the risk for a rabies outbreak in the country exists, given the imported human rabies cases in 2006. To prevent imported rabies, it is necessary to clarify the reservoir such as bats and the other wild animals and the infection cycle among them. Therefore, we surveyed the epizootic and epidemiologic situation of rabies and the diseases caused by lyssaviruses in the countries surrounding Japan and the endemic areas of the world, where the situation is not yet well known.

## Materials and Methods

Rabies virus and lyssavirus isolates were collected from wild animals such as bats and foxes in Brazil. Additionally, the epidemiologic survey of the viruses was conducted in northeast, northwest, and southern China. Viral RNA extracted from the virus isolates in southern China was analyzed phylogenetically.

## Results and Discussion

1) Cattle rabies in Brazil is derived from several regionally defined variants, which suggests that its geographic distribution is related to that of the vampire bat population.

2) The survey on the human rabies cases in the forest of northern Brazil showed that the cases were derived from vampire bat–related virus. At the same time, rabies derived from dogs was endemic to the same area.

3) Rabies virus isolated from foxes in northeast Brazil was phylogenetically different from the known dog rabies virus.

4) In southern China, where human and dog rabies is epidemic, rabies virus isolated from livestock was derived from dog rabies virus.

## Conclusions

1) Community-based control of vampire bat populations effectively prevented the epidemic of rabies typified by livestock rabies in Brazil. To clarify the epidemiologic background of the diversity of rabies derived from wild animals, an extended epidemiologic survey is necessary.

2) The control of rabies infection cycle among dogs and wild animals is imperative in areas where vaccination and detainment systems for domestic dogs are incomplete.

3) In the instance of the epidemic of human and dog rabies in southern China, where rabies in humans and dogs is detected frequently, countermeasures against dog rabies are important for preventing the disease.

### Reference

1. Sakai K, Ito T. An epidemiological study on the international surveillance of rabies and the diseases by lyssaviruses that are caused by wild animals. A study supported by Grants for Health Science from the Ministry of Health, Labour and Welfare of Japan [in Japanese]. 2007 [cited 2008 Apr 11]. Available from http://mhlw-grants.niph.go.jp/index.html
